# War Prices Depriving Babies of Milk

**Published:** 1917-12

**Authors:** 


### WAR PRICES DEPRIVING BABIES OF MILK.

Decreases roeported from New York and Chicago and New England Cities in the amount of milk now being consumed by families with young children have led the Federal Children's Bureau to emphasize its imperative necessity in the diet of babies and young children.

Dr. Grace L. Meigs, the Director of the Bureau's Child Hygiene Division, in commenting on the danger of such a decrease to the health of children today, said "Milk is the one food that all young children must have if they are to be strong and healthy. Whole milk is rich in the elements without which the child's growth ceases and his health is impaired; indeed there is no food which can supply as well the needs of the growing child.

"There is no substitute for milk in the diet of babies and young children. Yet the increase in its price is so startling that, as the reports the Bureau receives show, many mothers are economizing on milk. Young children can not get the nourishment they require from the would-be milk substitutes given them. Patent foods which do not themselves contain milk and are not intended to be mixed with milk are so lacking in the essentials of healthy development that we must expect children fed on them instead of on milk to be weakly and ailing. Plainly very great harm is done young children by giving them tea and coffee to take the place of milk which is really a complete food; it is giving them more stimulant to replace their best food."

Since the price of milk went up to 14 cents a quart, tea and coffee have been substituted for milk by more than half of the 2,200 families—all with children under six—included in the study of the effect of the increased price of milk just made in New York City by the Mayor's Committee on Milk, the City Department of Health and the Association for improving the condition of the poor. One hundred and twenty families have stopped taking milk entirely, in 25 of these there are babies under one year old. All of the 2,200 families have young children, but nearly half are taking from one-fourth to one-half less milk than before the price went up. Yet even before the larger price decreased the amount of milk they bought these families were getting but little more than half the amount of milk which experts on children's diets say they need.

In Chicago as well as New York the rise in the price of milk has forced down the amount purchased. A dealer there reports that while he distributed on an average 4,400 quarts of milk a day in September, on October 3 with the price increased, he distributed only 2,500 quarts.

In New Haven, Bridgeport and other Connecticut towns milk delivered at the station is sold wholesale at 8 cents a quart. It retails as high as 15 cents a quart. In Waterbury, when the price was raised from 12 to 15 cents a quart the sale was so greatly reduced that the price has been dropped back to 14 cents.

